# Virtual Reference Environments: a simple way to make research reproducible

**DOI:** 10.1093/bib/bbu043

**Published:** 2014-11-28

**Authors:** Daniel G. Hurley, David M. Budden, Edmund J. Crampin

**Keywords:** reproducible research, virtual environments, open source

## Abstract

‘Reproducible research’ has received increasing attention over the past few years as bioinformatics and computational biology methodologies become more complex. Although reproducible research is progressing in several valuable ways, we suggest that recent increases in internet bandwidth and disk space, along with the availability of open-source and free-software licences for tools, enable another simple step to make research reproducible. In this article, we urge the creation of minimal virtual reference environments implementing all the tools necessary to reproduce a result, as a standard part of publication. We address potential problems with this approach, and show an example environment from our own work.

Replication of results is a central tenet of science; the idea that a meaningful result should be able to be replicated, and that publication should describe it in enough detail for this to be possible, motivates a large part of the basic activity of science. Although the degree to which this is possible for large experimental results is under debate [1], it is reasonable to say that demonstrating and promoting replication of a result is valued by scientists.

However, reproducing computational results is still difficult and time-consuming for journal reviewers and readers. Problems with replicating computational results can spring from a variety of places: dependency problems with tools, libraries or operating systems and availability of appropriate data. Even when a result can be replicated, the time taken to set up and configure a tool on another system can be significant. Web-based tools can mitigate this, but they often obscure the technical details of implementation, meaning that it can be hard to rely on them to give the same result at different times. In this journal, Smith, Ventura *et al.* [2] have given a comprehensive summary of these issues applied to analysis of mass spectrometry data, although much of what they say is generally applicable to bioinformatics and computational biology. Minimum information standards for reproducible experiments have been discussed and publically available for some years (e.g. MIAME [3], MIABi [4]), although much recent discussion on the practical implementations of reproducibility frequently takes place on the Internet and in blogs rather than in peer-reviewed literature (e.g. http://ivory.idyll.org,http://recomputation.org,http://bioinformaticszen.com/)

The general concept of ‘reproducible research’ encompasses a number of different types of activity, and different strands of effort have evolved to support these activities. One sense of ‘reproducibility’ is facilitating ‘re-implementation’: making a clear and comprehensive ‘recipe’ of the ingredients and processes required to obtain a general result. Approaches supporting this sense include web-based workflow engines like Taverna [5] or Galaxy [6], ‘literate programming’ toolsets like Sweave for R [7] or iPython [8] standardized preconfigured environments like BioLinux [9] and cloud-based virtual environments [10].

Another sense of ‘reproducibility’ is ‘recomputability’ or ‘replicability’: making a ‘snapshot’ of a result presented in a publication which can be easily verified and explored by readers and a scientific community. Such a ‘snapshot’ is a permanent, accessible and archivable record of published work which does not depend on external data or code sources.

In our opinion, both the ‘recipe’ and the ‘snapshot’ sense of reproducibility are valuable activities, and in bioinformatics and computational biology, there is a simple step we can take to facilitate direct replication of results, which has only recently become possible because of increases in bandwidth and disk space, and the prevalence of open-source and free-software licences for bioinformatics tools and environments.

## A simple step to improve reproducibility

We propose that every publication presenting computational results should aim to have a ‘virtual reference environment’ accompanying the publication. This reference environment should be a minimal implementation of the software stack required to reproduce some or all of the computational part of the results. For a desktop tool, this will be an operating system, libraries, tools and data. By downloading this image and hosting it in a virtual machine monitor such as VMWare (http://www.vmware.com) or VirtualBox (https://www.virtualbox.org), readers and reviewers can immediately reproduce and investigate results, with minimal configuration effort. Many researchers now also have access to institutional or national ‘cloud’ environments, and a virtual reference environment can be easily deployed to one of these.

Using open-source and free software, such a reference environment is now technically straightforward to produce and distribute; it does not require specialized software development or system administration knowledge beyond the skill set of a typical bioinformatics researcher. If a result uses open-source or free software operating systems and tools, there are step-by-step tools available to create a snapshot of a working environment (e.g. Ubuntu Builder (https://launchpad.net/ubuntu-builder) or Relinux (https://launchpad.net/relinux))

To prove this principle, we have created such a reference environment for the accompanying publication ‘Predictive modeling of gene expression from transcriptional regulatory elements’ [11], and we have made this available at the SourceForge repository for the code presented in this publication (http://sourceforge.net/projects/budden2014predictive/files/latest/download). This reference environment was constructed using a minimal installation of Lubuntu, a lightweight Linux operating system derived from the popular Ubuntu distribution, delivered as an ISO image of ∼600 Mb. It includes all the software and tools necessary to replicate the results and figures presented in the accompanying paper. We have included scripts for figure generation so that motivated readers can go from source data directly to the figures presented in the manuscript itself.

## Potential obstacles

Although we believe strongly in this principle, we see some obstacles to its adoption, and we describe them below:

**Availability of data or code:** some data are confidential, embargoed or restricted in access. Some code is covered by proprietary licences; perhaps a result is only available using a commercial operating system like Microsoft Windows, or it requires a proprietary programming language.

**Licensing and distribution:** use of an environment requires acceptance of all the licence agreements associated with the entire software stack required to reproduce the result.

**Specific architecture requirements:** some results may require specialized hardware such as multiprocessor systems, or proprietary architectures.

**Problems with the scale of a result:** some results may require significant computational resources to replicate, and may require some days or weeks to complete.

**Problems with image size:** The image for a reference environment may be a large file (∼500 Mb+), but it is no larger than many types of raw data, and is considerably smaller than some (e.g. next-generation sequencing data). Ideally an environment should include all the data required to replicate some or all results in a publication, but where this is not realistic, readers may need to download data separately, as commonly happens now from data repositories.

**Issues of curation:** If a publication contains errors, the virtual reference environment accompanying a publication may contain corresponding errors in the data or code, and if the environment is downloaded, those errors may persist even if the publication is altered or retracted. To manage this, we suggest that the reference environment should only be used for the purpose of replicating the results in the publication; it is not intended as a general-purpose computational biology environment. Furthermore, all output from the reference environment should include citation information, and refer users back to the DOI or other identifier for the publication.

These obstacles are all genuine, but we feel that it is not at all a bad thing for barriers to replicating a result to be clearly stated as part of a publication. In this case, a reasonable approach would be to explain which results can be easily replicated in a reference environment, and which cannot, and for what reasons. Even a simple test implementation running a ‘toy’ problem in a reference environment has considerable value if it is an example of the software correctly set up and configured.

In the extreme situation that no aspect at all of a publishable result can be replicated in a reference environment for the reasons described above, it is valuable for that also be clearly stated. Although this situation is quite possible, we believe that it is not the norm in bioinformatics and computational biology, and it does not reduce the value of providing a reference environment for the majority of cases where it is not so.

In truth, all of the barriers to making and distributing a reference environment are actually barriers to reproducing the research itself; where these barriers exist, they should be acknowledged in a publication.

## Conclusion

We stress that we do not intend here to disadvantage or criticize researchers who use proprietary tools, or who work with data that are not freely distributable; both of these are unavoidable, and in fact we do both in our own work. The open acknowledgement of barriers to replication that we propose confers a reciprocal responsibility on reviewers and readers to assess these barriers in a measured and sensible way. Where they do exist, though, we undertake to state this in a clear and upfront manner, and we urge others to do the same.

Our proposal for providing a virtual reference environment for each publication is straightforward and within the resources of all researchers. Producing such an environment supports recomputation/replication, and serves an archival purpose for complex results, but it also promotes ‘transparency’ of reproducibility: the awareness of which aspects of a result are simple to reproduce, and which are not. We would like to see such an approach become a standard supported by journals, and as a possible step towards a general ‘charter for reproducible research’ in bioinformatics and computational biology, and we welcome discussion with the community on these matters.

**Key Points**

- Reproducibility of results is accepted as important in bioinformatics, but there are no standard approaches across all platforms, operating systems, data sets and languages
- Small differences between systems can make tools behave differently or not at all in one environment compared to another. This means that reviewers and readers may not be able to replicate a published result, despite genuine and appropriate effort by authors
- There is a simple solution to this for open-source or free-software licenced tools: create a minimal virtual environment which replicates the core results of a publication. Such an environment is a permanent ‘snapshot’ of tools and results and acts as a reference point for replication, troubleshooting and change management
- We have done this for results published in Briefings in Bioinformatics, and welcome discussion and feedback on our approach. We are happy to work with other researchers in the bioinformatics community to develop similar reference environments for their work

## Funding

This work was supported by an Australian Postgraduate Award [D.M.B.]; the Australian Federal and Victoria State Governments and the Australian Research Council through the ICT Centre of Excellence program, National ICT Australia (NICTA) [D.M.B., E.J.C.]; and the Australian Research Council Centre of Excellence in Convergent Bio-Nano Science and Technology (project number CE140100036) [E.J.C.]. The views expressed herein are those of the authors and are not necessarily those of NICTA or the Australian Research Council.
